# Signal detection analysis of blindsight in monkeys

**DOI:** 10.1038/srep10755

**Published:** 2015-05-29

**Authors:** Masatoshi Yoshida, Tadashi Isa

**Affiliations:** 1 Laboratory of Behavioral Development, Department of Developmental Physiology, National Institute for Physiological Sciences, National Institutes of Natural Sciences, 444-8585, Okazaki; 2 School of Life Science, the Graduate University for Advanced Studies (SOKENDAI), Hayama, 203-0193, Japan

## Abstract

Macaque monkeys with a unilateral lesion in V1 have been used as an animal model of blindsight. While objective proof of blindsight requires that the two aspects of blindsight (residual forced-choice localization and attenuated yes-no detection) should be tested under identical stimulus conditions using bias-free measures of sensitivity, these have not been attained in studies of nonhuman primates. Here we tested two macaque monkeys with a unilateral V1 lesion with two saccade tasks using identical stimuli: a forced-choice (FC) task and a yes-no (YN) task. An analysis based on signal detection theory revealed that sensitivity in the FC task was significantly higher than that in the YN task. Such dissociation of sensitivity between the two tasks was not observed when near-threshold visual stimuli were presented in the normal, unaffected hemifield. These results suggest that the V1-lesioned monkeys resemble the well-studied blindsight patient G.Y., whose visual experience per se was completely abolished.

Blindsight occurs in some patients with damage in the primary visual cortex (V1). These patients suffer from a loss of visual awareness in their contralateral hemifield. However, they are able to point to the visual stimulus when they are forced to guess its location^1^. Blindsight is an intriguing phenomenon for the study of consciousness because it provides a rare occasion where conscious awareness of salient visual stimuli can be dissociated from other aspects of visual information processing. In addition, blindsight has clinical importance because the restoration of visual function in the form of blindsight may improve the quality of life in hemianopic patients^1^. Because of this scientific and clinical importance, the development of an animal model of blindsight is a key strategy to expand our understanding of this condition. Previous studies showed that macaque monkeys with a unilateral lesion of V1 exhibit residual visual processing as demonstrated by key press, reaching or saccadic eye movements^2^,^3^,^4^,^5^,^6^,^7^. These studies showed that the lesioned monkeys were able to discriminate or localize the visual stimuli presented in the contra-lesional visual field in a forced-choice (FC) task. A more difficult question is whether the monkeys are aware of the visual stimuli. A study by Cowey and Stoerig^5^ showed that V1-lesioned monkeys behave as if they are not aware of the stimuli in a ‘yes-no’ (YN) task in which the monkeys were asked to report the presence or absence of the stimuli. Since “this paradigm-dependent dissociation is reminiscent of patients with blindsight who claim that they do not see the stimuli they can nonetheless respond to in their hemianopic field”^5^, they conclude that the monkeys demonstrates “blindsight rather than degraded vision”^5^. Indeed, when the identical task was subsequently used to test human blindsight subjects, a similar dissociation was reported^8^. However, their study has been criticized in terms of the experimental design they had used^9^,^10^. These criticisms can be summarized as follows: 1) The stimulus conditions in the FC task and the YN task were not identical; 2) the two tasks were not performed during the same session; 3) no proper control condition, such as the normal, near-threshold condition^11^,^12^, was performed; and 4) decision bias based on signal detection theory^13^,^14^ was not considered.

Here we revisited the issue of visual awareness in monkeys with V1 lesion by using the FC task and the YN task in ways that overcome these deficiencies. 1) Identical stimuli were used in the two tasks. 2) Both tasks were performed during the same session. In the FC task, a visual saccadic target (ST) was presented in one of two positions in either the contra-lesional or ipsi-lesional hemifield. In the YN task, an additional trial condition, trials without saccadic target (ST−), was intermixed with trials with saccadic target (ST+) where the visual stimuli were identical to the FC task. 3) By comparison the FC and YN task results with the results of trials using near-threshold stimuli in the normal unaffected hemifield, we obtained evidence of blindsight in monkeys that is specific to the V1 lesion rather than near-threshold vision. 4) Finally, we introduced analyses based on signal detection theory and have drawn empirical ROC curves, thereby succeeded in evaluating the animals’ sensitivity to the visual stimuli independent of decision bias. As a consequence, we have identified a behavioural profile in monkeys that resemble blindsight in human subjects, including the well-studied patient G.Y. (see Discussion).

## Results

### Training, lesion and recovery

**Figure 1:**
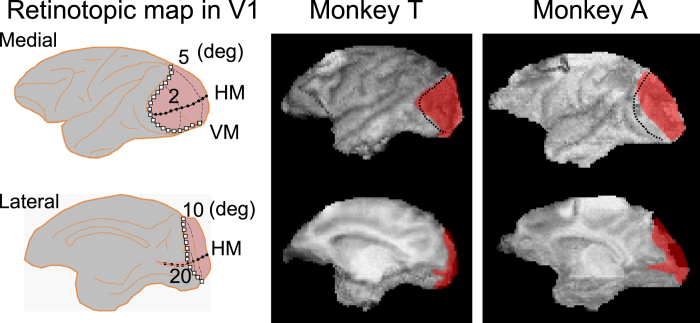
Extent of lesion. Left: The retinotopic map in V1 drawn on a macaque brain based on published literature. Middle and right: 3D images of the brain after the V1 lesion for two monkeys were reconstructed from the MR images. The lesion site, estimated from the MR images, is drawn in red. The dotted lines denote the border between V1 and V2. Top, medial view. Bottom, lateral view. VM, vertical meridian; HM, horizontal meridian.

Two Japanese macaque monkeys were trained with the FC task and the YN task before the lesion. Both monkeys attained a > 95% correct ratio. Then the left V1 was surgically removed from the monkeys (see Methods). The extent of lesion was assessed anatomically and functionally as described previously^7^. Briefly, structural imaging reconstruction using 3T MR (Allegra, Siemens) indicated that the lesion covered all of the contra-lesional visual hemifield from 5–25° eccentricity in both monkeys^7^ (Fig. 1). Using a visually guided saccade task with a five-alternative forced choice condition, we showed that the threshold for luminance contrast was significantly increased in the contra-lesional visual hemifield from 5-25° eccentricity in both monkeys^7^. The correct ratio for a visually guided saccade task with two alternative forced choices decreased to near chance level just after the lesion and recovered to >90%, and became stable at approximately 8 weeks after the lesion^7^.

### Comparison of performances in the two tasks

**Figure 2:**
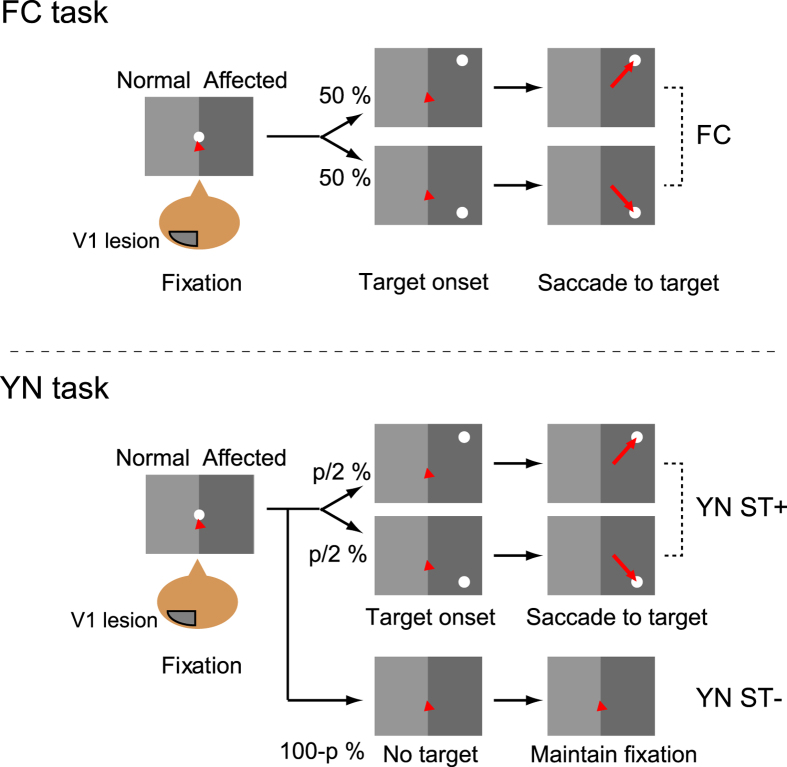
Behavioural tasks. Top, forced-choice (FC) task. After a fixation period, a saccadic target appeared in one of the two positions in one of the hemifield. Trials in which stimuli were presented in different hemifields were in different blocks. Bottom, Yes-no (YN) task. The ST+ condition (“YN ST+”) was identical to the FC task. Trials without a saccadic target (ST− condition) were randomly interleaved. The ratio for ST+ condition (p %) was fixed as 30% in the results for Fig. 3 and varied across trial blocks in the results for Fig. 4.

**Figure 3:**
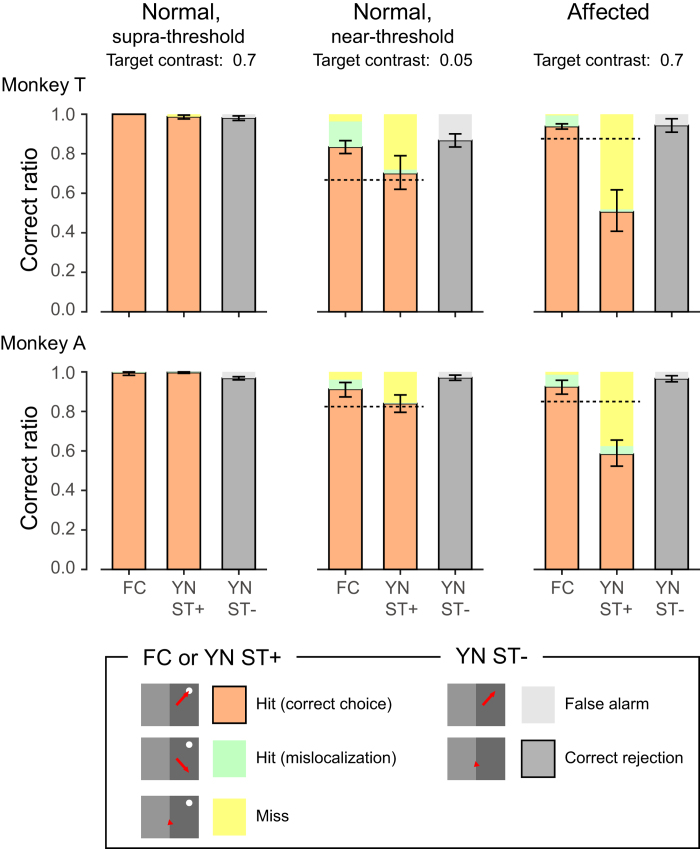
Performance of FC task and YN task. The bars with black edges indicate the ratio of correct, rewarded trials to the total trials. See the text for classification of trials according to the behavioural responses. FC, FC task. YN ST+, ST+ condition of YN task. YN ST−, ST− condition of YN task. Left panel, trial blocks in which supra-threshold stimuli (target contrast 0.7) were presented in the normal hemifield (“Normal, supra-threshold”). Middle panel, trial blocks in which near-threshold stimuli (target contrast 0.05) were presented in the normal hemifield (“Normal, near-threshold”). Right panel, trial blocks in which stimuli (target contrast 0.7) were presented in the affected hemifield (“Affected”). Probability of ST+ trials (“p” in Fig. 2) was fixed at 30%. Top, monkey T. Bottom, monkey A. Dotted lines: Performance of the YN ST+ condition (P_YN_) expected from that of the FC condition (P_FC_): P_YN_ = P_FC_ * 2–1.

**Figure 4:**
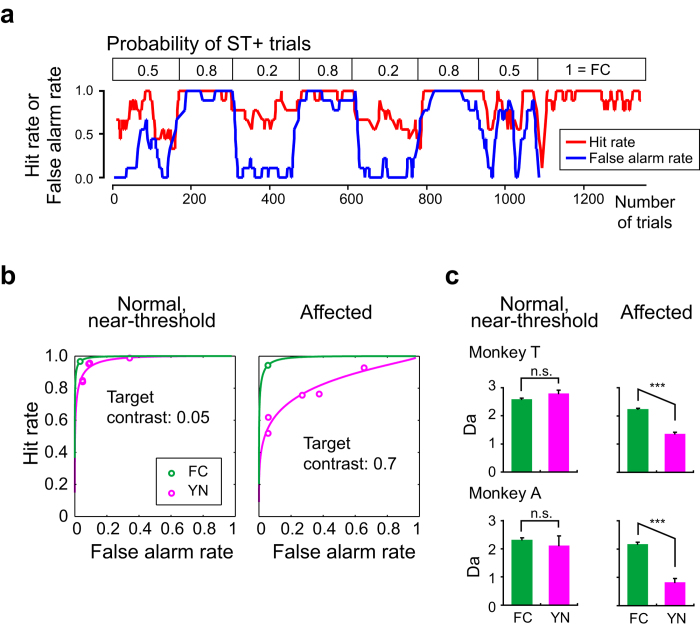
Behavior and analysis based on signal detection theory. **a** An example of a session in which the probability of ST+ trials were varied in different blocks (on average, 161.1 trials per blocks). The value for each point represents a running average of the adjacent nine trials. Red, hit rate. Blue, false alarm rate. “FC = 1” indicates a trial block for the FC task. Monkey T, 18 months after V1 lesion. **b** Empirical ROC curves for Normal, near-threshold condition (left) and for Affected condition (right). Magenta, data for YN task. Green, data for FC task. A circle indicates data point for each ST+ probability. Lines indicate fitted lines. For YN task (magenta lines), the fitting was based on the unequal-variance model. Monkey T. **c** Sensitivity, as expressed in Da, was plotted for FC and YN task and for two trial conditions and for monkey T (top) and monkey A (bottom). ***, p < 0.001; n.s., not significant.

**Figure 5:**
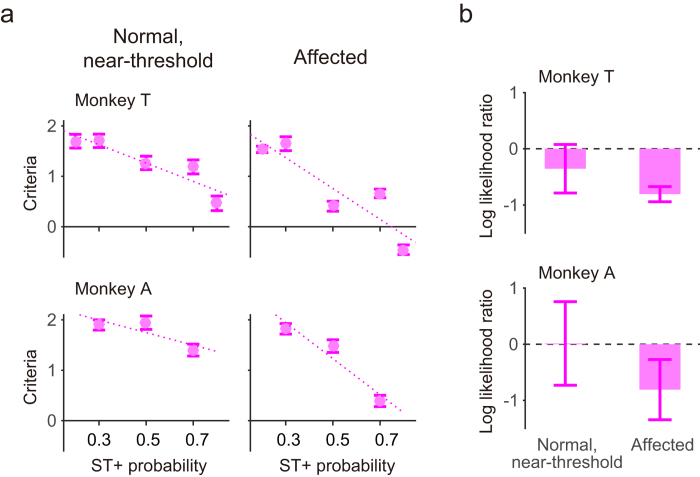
Decision bias in the YN task. **a** The decision criterion for the YN task (with reference to the noise level in signal detection theory) was plotted across the ST+ probability. In all cases, the correlation coefficients were smaller than −0.81. The error bars indicate the standard deviation. **b** The log likelihood ratio at the criterion with 50% ST+ probability was plotted for both monkeys and two stimulus conditions. The value zero indicates optimal decision and the negative values indicate bias toward saccades over fixation. Error bars indicate the standard deviation.

**Figure 6:**
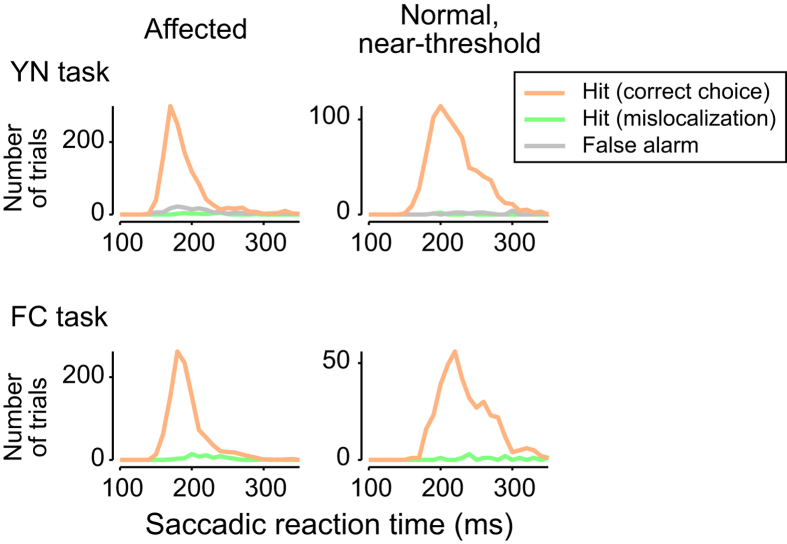
Saccadic reaction time. The distribution of saccadic reaction times for the two tasks and for the two stimulus conditions. See Fig. 2 for classification of response types. Monkey T. 10 ms bins.

Behavioural tests using the FC and YN tasks (Fig. 2) were performed after the lesion when performance of the visually guided saccade task had stabilized (16-18 months for monkey T and 23-24 months for monkey A). In the normal, unaffected hemifield, the correct ratio for the FC task was more than 95% in trial blocks where the saccadic target was presented in one of two possible positions for both monkeys (100% in monkey T and 99.5% in monkey A; ‘FC’ in the left panel ‘Normal, supra-threshold’ in Fig. 3). In the YN task, the experimental trials were classified into five categories on the basis of outcome. In the ST+ condition where the saccadic target was presented, possible responses were saccades to the correct position (‘Hit (correct choice)’), saccades to the incorrect position (‘Hit (mislocalization)’) and fixations maintained for more than 700 ms (‘Miss’). In the ST− condition where the saccadic target was not presented, possible responses were fixations maintained for more than 700 ms (‘Correct rejection’) and saccades to potential target positions (‘False alarm’). The trials with the ST+ condition and the ST− condition were intermixed randomly and the ratio of ST+ condition among all YN trials was fixed at 30% in the data for Fig. 3 (which was varied in the data shown in Figs. 4, 5, 6). In both the ST+ and ST− conditions, the ratio for the correct response (“Hit (correct choice)” and “Correct rejection”, respectively) was higher than 90% in both monkeys (98.7% and 98.1% in monkey T and 99.6% and 96.7% in monkey A, respectively; “YN ST+” and “YN ST−“ in the left panel “Normal, supra-threshold” in Fig. 3). These results suggest that the monkeys were able to perform both tasks (FC and YN tasks) that they were trained before the lesion, and that they were able to change their behavior according to the task rule for each trial block.

In other trial blocks, the saccadic target was presented in the contra-lesional, affected hemifield. The correct ratio for the FC task was >90% in both monkeys (93.8%, 1135/1210 in monkey T and 92.4%, 197/213 in monkey A; “FC” in the right panel “Affected” in Fig. 3). Since the 95% credible interval of the correct ratio was 92.0-94.7% in monkey T and 88.7-95.3% in monkey A, the correct ratio was significantly larger than the chance level (50%). These findings, indicated that the monkeys were able to exhibit residual vision as reported previously^2^,^3^,^5^,^7^. On the other hand, the correct ratio for the ST+ condition of the YN task (“Hit (correct choice)”) was 50.6% (41/81) for monkey T and 58.3% (115/197) in monkey A (“YN ST+” in the right panel “Affected” in Fig. 3). Since the 95% credible interval for the correct ratio was 40.7-61.7% for monkey T and 52.2-65.4% in monkey A, these values were significantly lower than the correct ratio expected from the FC task (87.6% for monkey T and 84.8% for monkey A; the dotted lines in the right panel “Affected” in Fig. 3). Thus, 90% correct in the FC task suggests that the subject identified the saccadic target in 80% of the trials and selected the correct target in 10% of the trials by chance (50% of the 20% trials in which the subject did not identify the target). These results indicate that in about half of trials in the ST+ condition of the YN task, the monkeys performed as if it was the ST− condition of the YN task. These results show that the monkeys failed to detect the presence of the saccadic target (YN task), yet they succeeded in localizing the saccadic target in the forced choice condition (FC task). These results also provide an intriguing speculation that the monkeys are not “able to see” the saccadic target, which is an analogous observation to the blindsight patients as reported previously^12^.

Here we report a performance dissociation between the ability to localize a visual stimulus in the forced choice condition (FC task) and the ability to detect the presence of the same stimuli (YN task). However, such dissociation can arise in the normal, near-threshold condition as reported previously^11^. To exclude this possibility, the same monkeys were tested with the same tasks when the saccadic targets had low luminance contrast (0.05 in Michelson contrast), and were presented to the normal, unaffected hemifield (the middle panel “Normal, near-threshold” in Fig. 3). In the FC task, the correct ratios were 83.3% (411/493) in monkey T and 91.2% (187/205) in monkey A, which were comparable to the correct ratios of the performance of the FC task in the affected hemifield (the left panel “Affected” in Fig. 3). On the other hand, the correct ratio for the ST+ condition of the YN task was 70.0% (70/100) for monkey T and 83.9% (209/249) in monkey A (“YN ST+” in the middle panel in Fig. 3). Since the 95% credible interval for the correct ratio was 62.0-79.0% for monkey T and 79.5-88.3% in monkey A, these values were not significantly lower than the correct ratio expected from the FC task (66.6% for monkey T and 82.4% for monkey A; the dotted lines in the middle panel in Fig. 3). These results suggest that the dissociation of performance in the two tasks in the affected contra-lesional hemifield was specific to the V1 lesion, rather than some general phenomenon found in normal vision when the stimuli are near-threshold.

### Behavioural test and analysis using signal detection theory

Our YN task has been used in human subjects to report the presence/absence of the stimuli. However, for the monkeys it is essentially an economical decision to obtain juice reward. The ratio of ST+ trials to all trials can affect the monkeys’ decision whether to make a saccade or maintain fixation. It is important, therefore, to compare the sensitivity in the two tasks based on signal detection theory.

We varied the ratio of ST+ trials to all trials (ST+ ratio) in blocks of >100 consecutive trials within a session and calculated the hit rate (“Hit” trials out of “Hit” trials and “Miss” trials) and the false alarm rate (“False alarm” trials out of “False alarm” trials and “Correct rejection” trials). In one example session, the monkey performed the YN task adaptively with trial blocks having different ST+ ratios, followed by the FC task (Fig. 4a; monkey T, 8 months after the lesion). We plotted the empirical receiver-operating characteristics (ROC) curves for all of the data in the sessions with varied ST+ ratio (Fig. 4b; 6043 trials for monkey T and 4452 trials for monkey A). The ROC curve was fitted for the stimuli in the affected hemifield for the YN task by a magenta line (“Affected” in Fig. 4b) and for the FC task by a green line. The magenta line is not symmetric for the y = −x axis, suggesting that the equal-variance model in term of signal detection theory was not valid in this case. Thus, we chose Da instead of d’ to evaluate sensitivity^15^. The Da for the YN task was significantly smaller than for the FC task in both monkeys (Z = 13.2, p < 0.001 for monkey T and Z = 8.9, p < 0.001 for monkey A with Bonferroni correction; “Affected” in Fig. 4c)^16^. All Da’s for the YN task and FC task were significantly higher than zero (Z > 6 and p < 0.001 for all Da’s)^16^. This is strong evidence for dissociation of sensitivity in the FC and YN tasks, even when the monkeys’ bias based on their economic decision was taken into account.

As shown in Fig. 3, we performed the same behavioural test and analysis for the normal, near-threshold condition. In separate sessions (1811 trials for monkey T and 2660 trials for monkey A), the saccadic target was presented in the normal, unaffected hemifield with the visual stimulus having a near-threshold luminance contrast (at ~0.05 in Michelson contrast). The ROC curves were fitted (Fig. 4b) and the Da was calculated (Fig. 4c). In both monkeys, the Da for the YN task was not significantly different from that of the FC task (Z = 0.5, p = 0.28 for monkey T and Z = −1.66, p = 0.95 for monkey A with Bonferroni correction; ‘Normal, near-threshold’ in Fig. 4c)^16^. The analysis based on signal detection theory therefore supports our conclusion from Fig. 3 that the difference in the affected hemifield performance in the two tasks was specific to the V1 lesion, as opposed to reflecting general characteristics of normal vision when presented with near-threshold stimuli.

### Evaluation of decision bias in the YN task

Previous reports showed that human blindsight patients^12^,^17^,^18^ exhibit not only a difference in sensitivity in the YN task and the FC task but also in decision bias so that their decisions about the detection of a visual stimulus are not optimal, as is the case with normal vision. We, therefore, examined decision bias in the YN task. The decision criterion for the behavioural data described above was calculated and plotted across the ST+ probability (Fig. 5a). Consistent with the representative example in Fig. 4a, the monkeys adopted a low criterion (preference to saccade) when the ST+ probability was high, and a high criterion (preference to fixation) when the ST+ probability was low. In all four conditions, the correlation coefficients for the criterion and ST+ probability were less than −0.82.

Since the decision criteria were calculated with reference to the noise level, as specified by the signal detection theory, it was affected by the sensitivity (Da in Fig. 4c). To evaluate the decision bias in the YN task, we calculated the log likelihood ratio at the criterion with 50% ST+ probability (Fig. 5b). This is dependent on the sensitivity and applicable to the unequal variance model. When the near-threshold stimulus was presented in the normal hemifield, the log likelihood ratio was −0.35 (p = 0.20) for monkey T and 0.01 (p = 0.50) for monkey A. These values indicate that their decision-making was close to optimal. On the other hand, when the visual stimulus was presented in the contra-lesional, affected hemifield, the log likelihood ratio was negative (log likelihood = −0.80, p < 0.001 for monkey T and log likelihood = −0.80, p = 0.065 for monkey A). This indicates that the monkeys’ decisions were not optimal, and were biased toward making saccades (“yes” response) rather than maintaining fixation (“no” response). Thus, although the V1-lesioned monkeys appropriately adjusted their behavioural choice according to ST+ probability, their decisions were not optimal. This pattern of results is consistent with those obtained with human blindsight patients^17^,^18^,^19^.

### Saccadic reaction time

In the YN task, the monkeys maintained fixation for the ST− condition, rather than perform an explicit action, such as touching a permanently presented box on the screen^5^. It is therefore possible that a ‘miss’ response in our YN task simply reflected an inability of lesioned monkeys to make a decision within the imposed time window (700 ms after the offset of the fixation point). However, the distribution of saccadic reaction times (Fig. 6) shows that it was unlikely to be the case. In all conditions including the two tasks (FC and YN) and the two stimulus conditions (“Affected” and “Normal, near-threshold”), the reaction time for almost all the saccades was executed with less than 350 ms following the fixation point offset. Consequently, the probability of saccades with latencies of 350-700 ms was 1.3%, which is negligible. Thus, in our experiments, it is unlikely that the “miss” response was indicative of indecisive behaviour.

## Discussion

We tested two monkeys with a unilateral lesion of V1 and found that the sensitivity for stimuli in the affected hemifield was dissociated between the FC task, where the monkeys were forced to localize the stimulus position, and the YN task where the monkeys were asked to indicate the presence or absence of the stimulus. This dissociation was not observed when a low contrast stimulus was presented to their normal visual field. These results show that the observed dissociation is specific to the V1 lesion rather than representing a general characteristic of normal near-threshold vision.

Our results provide strong support for blindsight in monkeys by considering several important deficiencies that were not considered in previous studies^5^,^20^. First, the stimulus conditions in the FC task and the YN task were identical in our study. In the YN task of Cowey and Stoerig^5^, the monkey obtained its reward by pressing a permanently outlined large square to indicate a trial without visual stimuli. However, no large square was visible in their FC task. As we have shown previously in a free-viewing task^21^, the monkeys with a V1 lesion almost always made their first saccade of the movie clip exclusively to a salient stimulus in the normal, unaffected hemifield (Fig. S1D-E in Yoshida *et al.* 2012^21^). Thus Cowey and Stoerig’s result could therefore be explained by invoking spontaneous saliency-driven behavior. However, our study has shown that even when the stimuli were identical, the visual sensitivity in the FC and YN tasks differed. Second, in Cowey and Stoerig’s study^5^, the monkeys were tested with the YN task at several months after the testing with the FC task^20^. Thus, the possibility that their monkeys used completely different strategies with different sensitivities and thresholds for visual stimuli to solve the two tasks could not therefore be excluded. Our study tested the monkeys with the FC task and YN task during the same session, thereby excluding this possibility. Third, without comparison with normal, near-threshold conditions, the Cowey and Stoerig’s study^5^ could not exclude the possibility that dissociation of performance in the two tasks was not specific to the V1 lesion but could arise from normal, near-threshold conditions^11^,^12^. Fourthly, in Cowey and Storeig’s study^5^, the number of possible target positions in the FC task was different from that of the YN task. Thus, their observed performance of the monkeys could have been affected by decision bias. Our study explicitly manipulated the monkeys’ decision bias, and, consequently using analysis based on signal detection theory^19^ the sensitivity for the visual stimuli was evaluated independently of decision bias. Finally, we also note that we used saccade tasks and monitored eye position to make sure that the visual stimuli were precisely presented to the desired position on the retina.

Our analysis, which was based on signal detection theory, showed that the sensitivity in the FC task was significantly higher than that in the YN task, and the sensitivity in the YN task was greater than zero (i.e. chance level) (Fig. 4). These results are in close agreement with findings from the well-studied blindsight patient, G.Y^12^,^19^. Our finding of non-zero sensitivity in the YN task in V1-lesioned monkeys is also more consistent with human blindsight findings^12^,^19^ than the previous study of blindsight in monkeys^5^ is, where the correct responses in their YN task were virtually absent. Consequently, we conclude that the behavior of monkeys with V1 lesions resembles that of a well-studied blindsight patient, G.Y., whose visual experience per se was completely abolished. This conclusion is also supported by the results concerning decision bias, as we argued below.

We showed that the V1-lesioned monkeys exhibited a bias toward the “yes” response (saccade vs. fixation) in the YN task, but only when the stimulus was presented in the lesion-affected hemifield (Fig. 5). This suboptimal decision-making is consistent with our previous finding that the decision threshold for making saccades to stimuli in the affected hemifield is lower than for stimuli in the normal hemifield^7^. This conclusion was estimated from the distribution of saccadic reaction times and subsequent computational modelling of the decision process based on the diffusion model^7^,^22^. Our previous finding also indicated a bias toward making a saccade rather than maintaining fixation. Our findings in V1-lesioned monkeys are consistent with observations in a human blindsight patient whose difficulty in wagering is based on their confidence in visual detection^17^. Thus, we speculate that the V1-lesioned monkeys are similarly not confident in their appreciation of visual stimuli in their contra-lesional, affected hemifield. However, direct proof for this hypothesis would require the YN task to have a confidence judgement^23^,^24^ (type II task), rather than a manipulation of bias (type I task) as used in the current study.

Azzopardi^19^ reported that the human blindsight patient G.Y. showed a conservative bias for the “yes” response (meaning he has bias toward “no” response) in their YN task. Ko and Lau^18^ proposed that such a bias could be explained by an impairment of metacognition. At first, this might seem inconsistent with our present interpretation. However, there may be important differences in the task strategy used by the human patients and our monkeys. For humans, the YN response is an introspective report of awareness, while in the monkey experiments, it is an economical decision to obtain reward. Indeed, when G.Y. was asked to guess the presence of visual stimuli when he was not sure in the yes-or-guessing task, which is more similar to the monkey YN task, his conservative bias was abolished^19^. We also note that the monkeys’ bias toward the “yes” response could arise from an asymmetric design of responses, that is, saccades vs. fixation.

Our results have implications that may have a great impact on contemporary consciousness research. We found a clear dissociation of the sensitivity between the two tasks. Moreover, the sensitivity (Da) for the YN task was greater than zero, which has also be reported in human blindsight (G.Y)^12^. The standard psychological view assumes that non-zero sensitivity for the YN detection task reflects a retained form of visual awareness. However, this supposition is not straightforward in the case of blindsight. Some blindsight patients report a specific kind of awareness (“a feeling-of-something-is-happening”), an experience of the presence of the visual stimuli despite no phenomenal vision such as a shape or colour of the stimuli^25^,^26^. This experience generally occurs only when the visual stimuli is salient, and hence, has been called “type II” blindsight^1^. We therefore propose two possible interpretations of awareness in monkeys with blindsight in our study. One is that the non-zero sensitivity for YN detection indicates another type of blindsight so that guessing the presence of the target is greater than chance even without awareness. Another interpretation is that the non-zero sensitivity for YN detection was guided by a specific kind of awareness, “a feeling-of-something-is-happening” as in the type II blindsight of human patients. We cannot decide which is correct since this is a question about conscious experience in monkeys. All we can do is to speculate based on extrapolation from human findings. In humans, the dissociation of the sensitivity between the FC task and YN task was evident only when the stimuli were static^19^. When the stimuli were moving (moving bar or random dots), where the experience of “feeling-of-something-is-happening” is reported^25^,^26^, such dissociation was no longer observed^19^. Considering that we used static stimuli in the current study, the dissociation of the sensitivity between the FC task and YN task may signify a complete absence of awareness in V1-lesioned monkeys.

## Methods

### Animals

Two Japanese monkeys (*Macaca fuscata*; monkey A, male, body weight 9.0 kg and monkey T, female, body weight 6.5 kg) were implanted with scleral search coils^27^ and a head holder. All surgeries were performed under aseptic conditions as described previously^7^. Anaesthesia was induced by administration of xylazine hydrochloride (2 mg / kg, i.m.) and ketamine hydrochloride (5 mg / kg, i.m.) and was maintained with isoflurane (1.0-1.5%). All experimental procedures were performed in accordance with the National Institutes of Health Guidelines for the Care and Use of Laboratory Animals and approved by the Committee for Animal Experiment at National Institute of Natural Sciences. The monkeys were allowed to recover for more than 2 weeks before starting the preoperative training.

### Stimuli

Visual stimuli were presented on a CRT monitor (21 inch, Mitsubishi RD21GZ) positioned 28 cm from the eyes. Visual displays and data storage were controlled using computers running a real-time data acquisition system (Reflective computing, Tempo for Windows) with a dynamic link to Matlab (MathWorks). The CRT monitor was calibrated as described previously^7^. Luminance contrast of the targets was expressed as Michelson Contrast and was fixed as 0.7 except for the near-threshold condition in the normal, unaffected hemifield where the luminance contrast was fixed as 0.05. This contrast was chosen based on the psychometric function derived from our previous study (see Fig. 3 in Yoshida *et al.* 2008^7^). Background luminance was set at 1 or 3 cd / m^2^, because comparable values were chosen in neurophysiological studies that investigated the visual response of V1 neurons to stimuli presented in the natural blind spot in macaque monkeys^28^,^29^. In these studies, the effects of light scattering from the natural blind spot must be avoided.

### Preoperative training

The monkeys were placed in a primate chair with their heads in a fixed position and were trained to perform a visually guided saccade task with four possible targets for a liquid reward. Eye movements were recorded using the magnetic search coil^30^ with a resolution of 0.1 degree. Horizontal and vertical eye positions were sampled at 1 kHz. The FC task was trained first. At the beginning of each trial, the fixation point (FP) appeared at the centre of the screen, and monkeys were required to move their eyes to the FP. The duration of fixation was varied randomly between 400 and 1000 ms. When the eye positions deviated more than 1.5 degree from the FP, the trial was aborted. The saccadic target (a small spot of light 0.45 degrees in diameter) appeared in the peripheral visual field concurrently with the offset of the FP. Monkeys were rewarded with fruit juice if saccades were made less than 700 ms after offset of the fixation point and if fixation was maintained for 100-300 ms in the target window (size 2-3 degrees). Target eccentricity was fixed at 10 degrees. Target direction was either upper 30 degrees or lower 30 degrees for each hemifield. A small percentage of trials with saccadic reaction times less than 80 ms were considered to be trials with anticipatory saccades and were omitted from the analysis. Inter-trial intervals ranged from 1500 to 2000 ms. After learning the FC task with >95% correct performance, the YN task was introduced. In a separate session, the monkeys were trained with an ST− condition (equivalent to the YN task with 0% ST+ condition). In these trials, the targets were not presented after offset of fixation point and the monkeys were rewarded if they maintain fixation for 700 ms. Finally, trials with a saccadic target (ST+ condition) were then intermixed with ST− condition. Both monkeys attained >95% correct performance in this task before the lesion.

### Unilateral V1 lesion

The procedure for making the lesion has been described previously^7^. Briefly, the posterior half of the operculum, the dorsal and ventral leaf and roof of the calcarine sulcus and the most posterior part of the stem of calcarine sulcus were surgically removed by aspiration with a small-gauge metal suction tube under anaesthesia (isoflurane 1.0-1.5%). After surgery, the monkeys were given penicillin G (80 thousand units/day, i.m.) and cefmetazole (0.5 g/day, i.m.) as antibiotics and dexamethasone sodium phosphate (0.5 mg/kg, i.m.) to minimize brain edema.

### Extent of lesion

The extent of lesion was also confirmed as described previously^7^. Magnetic resonance images (MRIs) of the brains of these monkeys were acquired after the surgery (Siemens Allegra 3T; MPRAGE-3D; voxel size 0.82 mm × 0.82 mm × 0.81 mm). Then the extent of the lesions was established by reconstructing the 3D images of the brain (Fig. 1) onto templates which the estimated lesion site was drawn, using CARET software^31^. Based on the published literature^32^,^33^, we estimated that the small area of the spared cortex corresponds to either the foveal visual field (less than approximately 2.5 degrees in eccentricity) or the peripheral visual field (more than 25 degrees in eccentricity). Thus we conclude that the lesion was complete in the relevant area of the contra-lesional visual field used for the behavioural task (10 degrees in eccentricity).

### Postoperative training and behavioural test

Postoperative training was started 6 days (monkey A) or 21 days (monkey T) after the surgery, at which time the monkeys’ general behavior in the cage appeared normal. Initial recovery after the V1 lesion was assessed with the FC task as described previously^7^. After the recovery, the YN task was introduced again. The saccadic target was presented exclusively in the ipsi-lesional hemifield unaffected by the V1 lesion. Both monkeys quickly remembered the task and the performances for ST+ and ST− conditions were again >90% in both monkeys (The left panel “Normal” in Fig. 3). The monkeys were then tested in separate sessions with the YN task when the saccadic target was presented in the contra-lesional, affected hemifield, or with low luminance-contrast saccadic target presented in the ipsi-lesional, unaffected hemifield.

The behavioural test for signal detection theoretical analysis was conducted 16-18 months after the lesion in monkey T (6043 trials in 6 sessions) and 23-24 months after the lesion in monkey A (4452 trials in 4 sessions).

### Analysis of saccadic eye movements

The calibration procedure for saccade measurement has been described previously^34^. Localization of the target was evaluated by calculating the ratio of success trials among all trials (“success ratio”). The trial was considered successful when the monkeys made a saccade to the quadrant containing the target. Since the monkeys were trained to make accurate saccades as described previously^7^, directional errors for correct saccades were less than 15 degrees. The saccadic reaction time was defined as the interval between the target onset and the saccade onset. Saccades were detected when the peak velocity of the polar component exceeded 200 degrees/s. Then the onset time of the detected saccade was defined as the time point preceding the detected saccade at which the velocity exceeded 30 degrees/s. The end point of the saccades was defined as the spatial position at which the velocity of the saccade declined below 30 degrees/s after the saccadic onset. All of the analyses were conduced with Matlab 2014b (Mathworks).

### Analyses based on signal detection theory

To calculate the hit rate and the false alarm rate for each trial block with an ST+ ratio, the first 20 trials for each trial block were removed from analysis during which the monkeys were still adapting to the new ST+ ratio. To evaluate sensitivity, we chose Da, which is the sensitivity measure of the unequal variance model^15^,^35^. We also calculated the criteria (lambda) and the log likelihood ratio (beta), the latter of which is applicable to the unequal variance model^15^,^35^. The Da and log likelihood ratio were calculated with the software FitRoc: Parameter Estimation for Gaussian Signal Detection Model^35^. To compare the sensitivity between the two tasks, the sensitivity for the FC task was divided by the square root of 2, as specified by the signal detection theory. All of the statistical tests and analyses were conduced with Matlab 2014b (Mathworks) or JMP 11 (SAS Institute).

### Check for stray light

The standard procedure to exclude the possibility that light scattering may contribute to residual vision is to test the subjects’ ability to detect visual stimuli presented in the natural blind spot in the normal hemifield^36^,^37^,^38^. We previously confirmed that the monkeys used in this study were not able to use stray light to make correct saccade to the stimuli presented in the natural blind spot in the normal, unaffected hemifield (Supplemental fig. 4S of Yoshida *et al.* 2008^7^).

## Additional Information

**How to cite this article**: Yoshida, M. and Isa, T. Signal detection analysis of blindsight in monkeys. *Sci. Rep.* **5**, 10755; doi: 10.1038/srep10755 (2015).
